# Acute Spinal Extradural Hematoma and Cord Compression: Case Report and a Literature Review

**DOI:** 10.7759/cureus.11603

**Published:** 2020-11-21

**Authors:** Eslam Hussein Mohamed, Landric B Dsouza, Wael Abdelrehem Elnabawy, Khalid Bashir, Amr Elmoheen

**Affiliations:** 1 Emergency Department, Hamad Medical Corporation, Doha, QAT; 2 Medicine, Qatar University, Doha, QAT

**Keywords:** backache, cauda equina syndrome, bilateral limb weakness, epidural hematoma, antiplatelets

## Abstract

A 50-year-old Asian male presented to the emergency department with sudden onset of bilateral lower limb weakness preceded by lower back pain, which developed after lifting a moderately heavyweight. As the pain increased in intensity, the patient was transferred by ambulance to the emergency department, and en-route lost complete motor (0/5 power and absent reflexes) and sensory control over his lower limbs. The patient's medical history was significant for diabetes mellitus, hypertension, chronic kidney disease, and coronary artery disease with percutaneous coronary intervention in 2018 and 2019. He was taking dual antiplatelets (aspirin and clopidogrel) along with other medications. Magnetic resonance imaging (MRI) showed findings suggestive of acute and extensive extradural hematoma extending from the foramen magnum to the level of the fifth lumbar vertebra (L5), exerting severe mass effect on the cord with evidence of edema, most severe at the level from 7th to 10th dorsal vertebrae (D7-D10) vertebral level. The clinical features and the radiological findings confirmed the diagnosis of acute cauda equina syndrome.

This review is intended to promote awareness about a possible clinical correlation between the use of dual antiplatelet therapy as a risk factor of spinal hematomas and the cauda equina syndrome.

## Introduction

Spinal hematomas were first described as a clinical entity in the year 1850. These hematomas were discovered in the autopsies earlier, in 1682 by Tellegen [1]. Of these hematomas, spinal epidural hematoma (SEH) is a rare condition (0.1/100,000 patients per year) with male dominance of 3:1 and affecting mostly in the age group of 42-52 years olds [1]. The disease commonly occurs in association with factors such as trauma, coagulopathies, and epidural intervention [2]. Some spontaneous SEH cases were reported, which were idiopathic (one-third of the cases) and chronic. SEH with anticoagulant therapy is even less observed with the common presenting feature of acute radiating backache followed by features of nerve root compression immediately afterward [3,4]. The hematomas have been reported in cervicothoracic and thoracolumbar regions commonly, and the resultant cord compression has led to neurological deficits like quadriplegia and paraplegia in many reported cases [1,5,6]. Most of these cases showed a good prognosis when the immediate intervention was done, preferably a surgical one [2-4]. Although some cases even demonstrated good outcomes by giving medication when the surgical interventions were contraindicated or not available [1]. In cases where immediate intervention was not done, irreversible neurological deficits, i.e., cord compression syndrome or even death, were reported [2].

We report a case of a middle-aged male with multiple comorbidities, presenting with an acute history of backache, which was diagnosed as SEH and successfully treated with surgical intervention.

## Case presentation

The patient is a 50-year-old Asian male presented to the emergency department with bilateral lower limb weakness of three-hour duration and lower back pain, which developed after lifting a moderately heavy plastic box. As the pain increased in intensity, the patient was transferred to the emergency department by ambulance. En-route, he developed complete weakness and lack of sensation over his lower limbs. On arrival at the emergency department, the patient had lost both lower limbs’ motor power and sensation. The past medical history was positive for diabetes mellitus, hypertension, chronic kidney disease, and coronary artery disease. He also underwent percutaneous coronary intervention twice in 2018 and 2019, respectively. The patient was a social smoker, did not consume alcohol at the time, and had no previous similar episodes in the past. At the time of the presentation, he was on dual antiplatelets (aspirin and clopidogrel) for two years besides other medications, including lisinopril, bisoprolol, amlodipine, metformin for hypertension, diabetes, and coronary artery disease.

Physical examination

The vital signs showed a blood pressure of 177/111 mmHg, a pulse of 102 beats per minute, respiratory rate of 19 breaths per minute, and temperature of 36.9 °C. Appearance-wise, he was well-groomed, well-nourished, and overweight. The physical examination’s positive findings revealed decreased strength (0/5) in both lower limbs. The deep tendon reflexes were absent and the plantar reflexes were equivocal. Additionally, the patient had an absent sensation of pain and touch on both lower limbs, loss of proprioception below the inguinal ligament level, lax anal tone, and saddle anesthesia.

Investigations and diagnostic procedures

**Figure 1:**
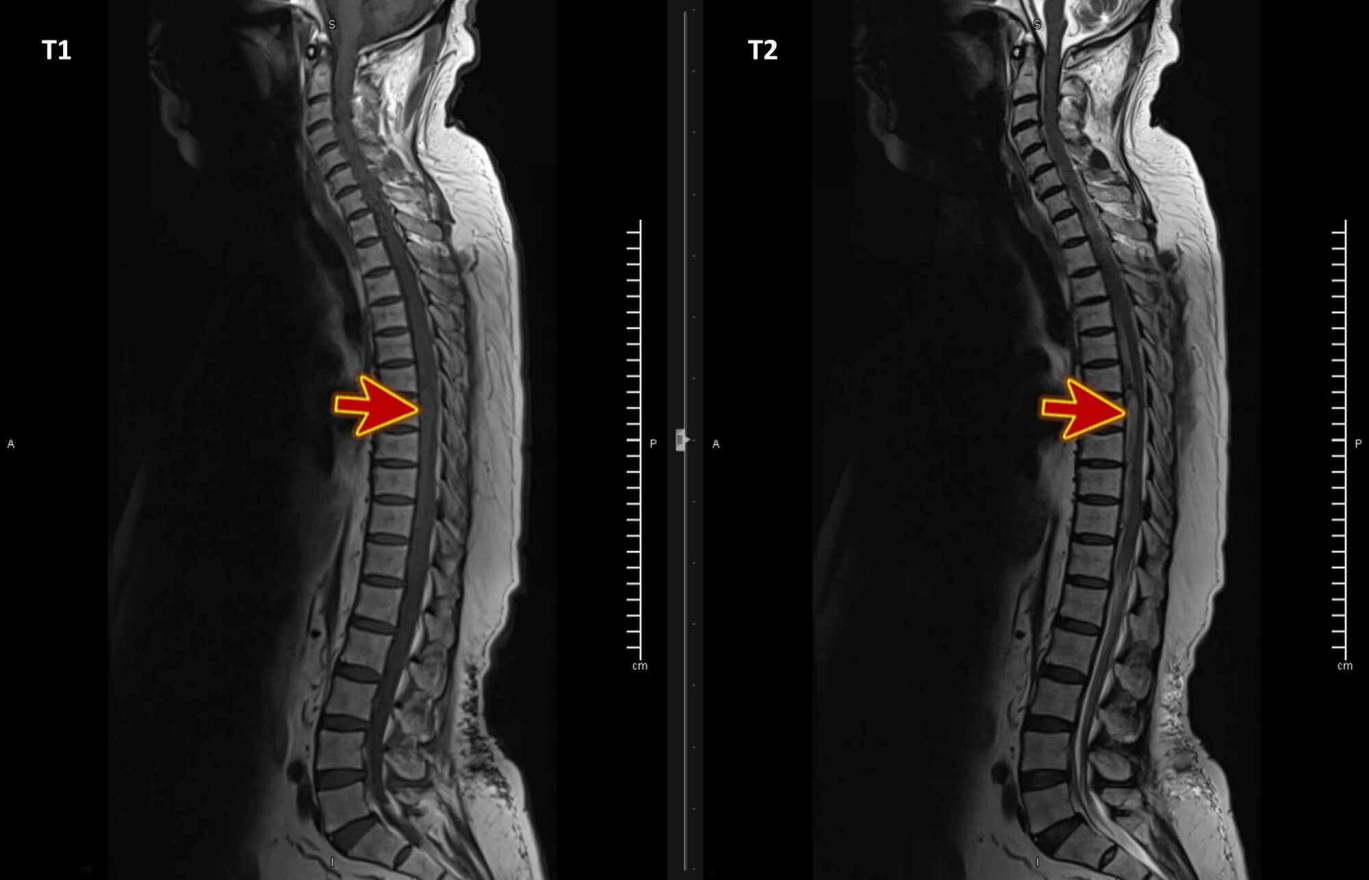
MRI T1WI and T2WI of the whole spines show extensive extradural hematoma (red arrows) extending from the foramen magnum to the fifth lumbar vertebral level (L5) MRI: magnetic resonance imaging, T1WI: T1-weighted image, T2WI: T2-weighted image.

**Figure 2:**
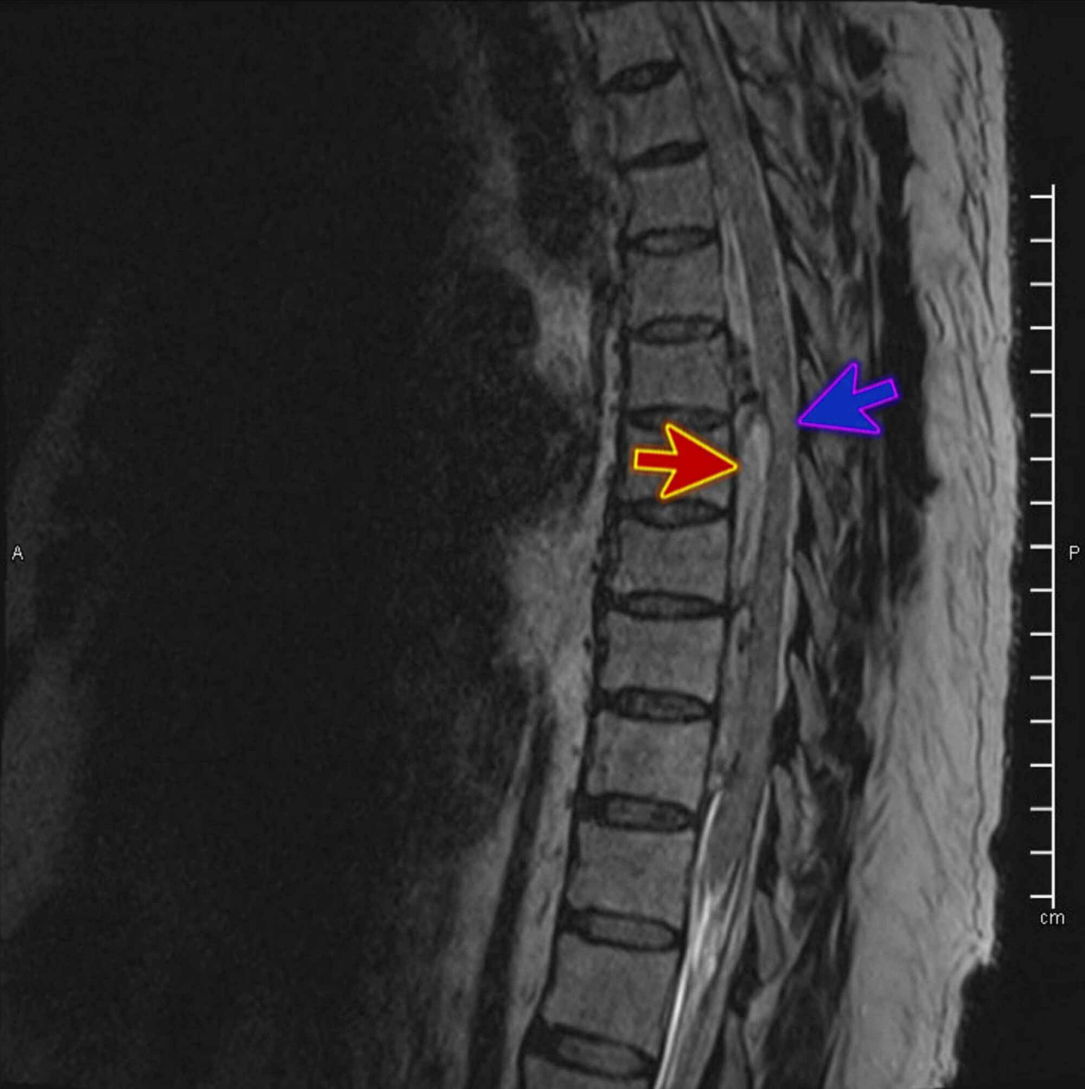
MRI T2WI sagittal view shows extensive extradural hematoma severe mass effect on the cord (red arrow) with evidence of edema (blue arrow), most severe at the level from the seventh to the tenth dorsal vertebra (D7-D10) MRI: magnetic resonance imaging, T2WI: T2-weighted image.

**Figure 3:**
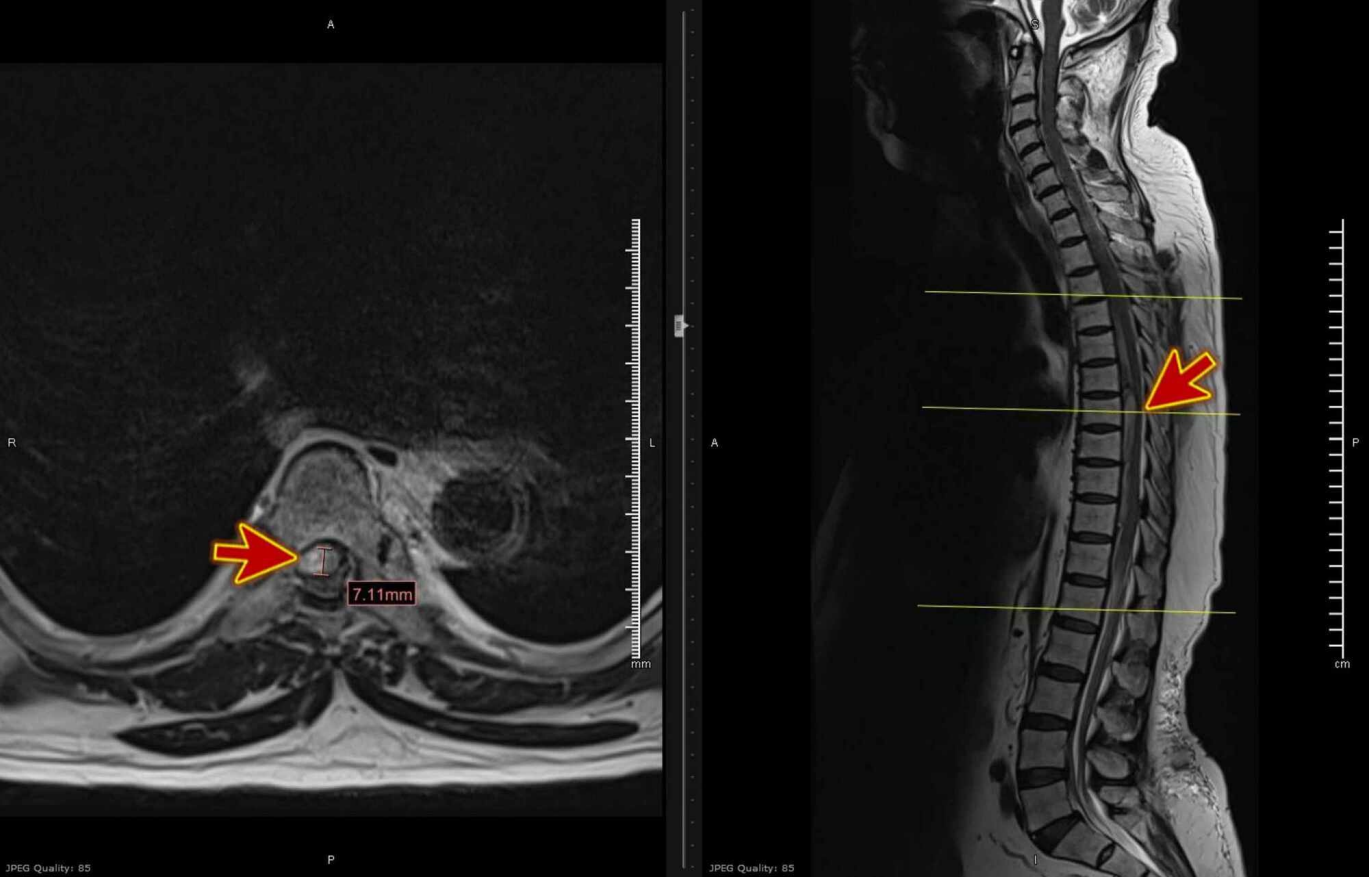
MRI T2WI axial view shows extensive extradural hematoma severe mass effect on the cord (red arrows), most severe at the level from the seventh to the tenth dorsal vertebra (D7-D10) MRI: magnetic resonance imaging, T2WI: T2-weighted image.

The patient’s blood workup, including a complete blood count and coagulation profile, yielded normal results. Magnetic resonance imaging (MRI; Figure 1) showed findings suggestive of acute and extensive extradural hematoma extending from the foramen magnum to the fifth lumbar vertebral level (L5), exerting severe mass effect on the cord with evidence of edema, most severe at the level from the seventh to the tenth dorsal vertebra (D7-D10; Figures 2 and 3) with high signal intensity was found on the T2 weighted image (T2WI). The vertebral bodies showed normal marrow signal intensity, preserved end-plates, and maintained height, while the sagittal fat-saturated T2 weighted imaging shows no evidence of marrow edema. The disks show maintained height and normal high signal intensity on T2WI with no significant disk bulge or herniation. Prevertebral signal intensity was normal.

Differential diagnosis

Considering the patient had bilateral lower limb weakness, loss of sensation, saddle anesthesia, and a lax anal tone, cauda equina syndrome was strongly considered. A secondary provisional diagnosis of spinal canal hematoma was made by taking into account the patient’s extensive past medical history and dual antiplatelets. MRI accomplished the definitive diagnosis of acute spinal extradural hematoma and cord compression.

Treatment

Urgent evaluation by the neurosurgery prior to MRI was done, and after the results of the radiological scan, an emergency operation involving a T5-T8 laminectomy was done with the evacuation of the epidural hematoma. The surgery was done within 12 hours of the onset of symptoms. Two drains were inserted, and the patient was transferred to the Surgical Intensive Care Unit (SICU) for further care.

Post-surgical outcome and follow-up

The patient showed little improvement post-operatively with a minimal flickering of toes bilaterally and generally poor muscle function in the lower limbs with sensation level intact up to the T12 level only. With a multi-disciplinary team discussion, it was decided in the patient’s best interest to continue to have regular occupational and physiotherapy sessions to improve lower limb strength and function for a goal of safe ambulation.

During his intensive care unit (ICU) stay, the patient developed severe aspiration pneumonia. He was intubated and on ventilatory support for a few days. The patient progressed to severe acute respiratory distress syndrome (ARDS), and he was supported by the extracorporeal membrane oxygenation (ECMO) device, but unfortunately, he remained in a critical situation that required high inotropic support and developed acute kidney shut down for which he underwent hemodialysis. Soon later, suddenly, he developed bradycardia and had a cardiac arrest. Cardiopulmonary resuscitation (CPR) was immediately initiated, but, unfortunately, he did not respond and declared dead due to cardiac arrest and multi-organ failure.

## Discussion

Spinal hematomas are of different varieties depending on the location of bleeding. It is usually classified as intradural (subdural), epidural, subarachnoid, or intramedullary [7]. Epidural hematomas are one of the frequently occurring (75%) types of spinal hematomas and occur when there is an accumulation of blood in the vertebral epidural space [4,7]. Historically, the epidural hematoma was initially reported in 1869 by Jackson, whereby he gave it a special terminology of “case of spinal apoplexy.” The first surgical evacuation of epidural hematoma via surgical intervention was successfully carried out in 1911 [7].

The etiology of spontaneous epidural hematomas is not very well established. Although some studies have postulated a probable correlation of epidural hematomas to different factors such as antiplatelet and anticoagulant use, blood coagulopathies, vascular malformations, tumors, pregnancy, and Paget’s disease, a vast majority of the cases (40-50%) remain idiopathic [2]. In our case, the patient had a history of diabetes mellitus, hypertension, chronic kidney disease, and coronary artery disease.

In 1955, Rader proposed that sudden and sharp rises in abdominal and thoracic pressures are intravascularly transmitted to the vessels’ intraspinal parts through the intervertebral foramen along the lateral spinal arteries and veins. Because of the spinal column and ligaments’ shielding effect, such a rapid increase in intravascular pressure cannot be neutralized by a simultaneous increase in spinal fluid pressure, leading to a large disparity between intravascular and extravascular pressure in the spinal canal and resulting in spinal vessel rupture [7-9]. In this case, the patient lifted a weight, which potentially triggered the hematoma formation. Non-spontaneous epidural hematomas, on the other hand, have many documented causes, and the commonest of them is post-spinal surgeries. These types of hemorrhages usually occur after 24-36 hours of spinal surgery [10].

In SEH, non-traumatic extreme back pain has been classically reported as the earliest and most marked symptom, accompanied by progressive neurological deficiency due to cord compression [1]; in some cases, there will be a typically related history of mild trauma whereby most of these lesions are found in the ventral region, but may occur in the dorsal, lateral, and often circumferential areas [8].

The classical symptoms exhibited in the patients presenting with epidural hematomas included sensory and motor deficits, sphincter dysfunction, Brown Sequard Syndrome, and even cauda equine syndrome [7]. The epidural hematoma symptoms in our patient were the complete loss of sensory and motor sensation, which was also reported in other patients with a similar issue [2].

Early diagnosis of the epidural hematoma is essential for a better outcome. There has been a case reported where the patient with epidural hematoma was diagnosed as a stroke based on clinical examination [2]. Although epidural hematomas have been diagnosed with myelogram and CT as well, the sensitivity and specificity of MRI are far superior [10]. MRI has been the established modality of investigation in patients with suspected spinal hematomas. It gives an obvious and detailed picture of the hematoma’s size and site and helps in the definitive diagnosis [7,11]. Our patient was also diagnosed with this radiological modality, along with the clinical signs and symptoms. It helped make a quick and early diagnosis, which is essential for the patient’s morbidity.

The concomitant use of anticoagulants in patients who were diagnosed with spontaneous epidural hematoma is also reported in some cases previously. In these cases, the most common hematomas with anticoagulant use are epidural, and subdural hematomas commonly occur in the lumbar region [1]. These patients used rivaroxaban, aspirin, and/or clopidogrel, which led to hematoma development [1,12]. Most of the patients had an international normalized ratio (INR) in the therapeutic range of 2.2 to 2.4 [1]. The duration of usage of these drugs is varying in diseased cases. Another similar case reported antiplatelet use for five years, which was slightly more than our patient who was using the medications for two years [2]. It is also noteworthy that anticoagulant therapy alone has not been reported to be the sole cause of epidural hematoma. Some studies have mentioned an added exertion or trauma, while others have noted increased pressure in the venous plexus to be the triggering factor for the disease [1].

Since the disease is very uncommon, there is no definitive treatment for this specific type of hematoma. Although interventions such as early emergency decompressive surgery performed within 36 hours have shown encouraging neurological outcomes when combined with physical therapy and rehabilitation [2,7,10,13,14]. Some clinicians have also vouched for less invasive endoscopic surgery for more chronic cases, but the definitive surgical intervention is yet to be established [10]. Furthermore, in some cases, non-surgical and conservative treatment is also recommended when either a surgical facility is not available or is contraindicated in the patient [1,15].

## Conclusions

Epidural hematomas are rare and serious hemorrhages with dangerous complications if not treated timely. As anticoagulants are increasing, clinicians should know this rare yet serious complication and closely monitor the patients. Also, the patients on anticoagulants should be monitored closely and advised to minimize activities like weight lifting, etc., as it can trigger the epidural hematoma. Furthermore, immediate surgical decompression is necessary to have better outcomes for the patient.
